# Investigating the cognitive capacity constraints of an ICU care team using a systems engineering approach

**DOI:** 10.1186/s12871-021-01548-7

**Published:** 2022-01-04

**Authors:** Jaeyoung Park, Xiang Zhong, Yue Dong, Amelia Barwise, Brian W. Pickering

**Affiliations:** 1 Department of Industrial and Systems Engineering, University of Florida, 482 Weil Hall, P.O. Box 116595, Gainesville, FL 32611-6595 USA; 2 Department of Anesthesiology and Perioperative Medicine, Mayo Clinic, Rochester, MN USA; 3 Division of Pulmonary and Critical Care Medicine, Mayo Clinic, Rochester, MN USA

**Keywords:** Cognitive function, Organizational decision making, Workload, Situational awareness, Systems approach, Electronic medical records

## Abstract

**Background:**

ICU operational conditions may contribute to cognitive overload and negatively impact on clinical decision making. We aimed to develop a quantitative model to investigate the association between the operational conditions and the quantity of medication orders as a measurable indicator of the multidisciplinary care team’s cognitive capacity.

**Methods:**

The temporal data of patients at one medical ICU (MICU) of Mayo Clinic in Rochester, MN between February 2016 to March 2018 was used. This dataset includes a total of 4822 unique patients admitted to the MICU and a total of 6240 MICU admissions. Guided by the Systems Engineering Initiative for Patient Safety model, quantifiable measures attainable from electronic medical records were identified and a conceptual framework of distributed cognition in ICU was developed. Univariate piecewise Poisson regression models were built to investigate the relationship between system-level workload indicators, including patient census and patient characteristics (severity of illness, new admission, and mortality risk) and the quantity of medication orders, as the output of the care team’s decision making.

**Results:**

Comparing the coefficients of different line segments obtained from the regression models using a generalized F-test, we identified that, when the ICU was more than 50% occupied (patient census > 18), the number of medication orders per patient per hour was significantly reduced (average = 0.74; standard deviation (SD) = 0.56 vs. average = 0.65; SD = 0.48; *p* < 0.001). The reduction was more pronounced (average = 0.81; SD = 0.59 vs. average = 0.63; SD = 0.47; *p* < 0.001), and the breakpoint shifted to a lower patient census (16 patients) when at a higher presence of severely-ill patients requiring invasive mechanical ventilation during their stay, which might be encountered in an ICU treating patients with COVID-19.

**Conclusions:**

Our model suggests that ICU operational factors, such as admission rates and patient severity of illness may impact the critical care team’s cognitive function and result in changes in the production of medication orders. The results of this analysis heighten the importance of increasing situational awareness of the care team to detect and react to changing circumstances in the ICU that may contribute to cognitive overload.

**Supplementary Information:**

The online version contains supplementary material available at 10.1186/s12871-021-01548-7.

## Background

An intensive care unit (ICU) is a complex and dynamic socio-technical system, which imposes a highly challenging work environment on care providers both physically and emotionally [1]. To deliver critical care, multidisciplinary medical professionals (e.g., intensivists, nurses, advanced practice providers, pharmacists, and respiratory therapists) collaborate as a hierarchical team and make team-based clinical decisions [2, 3]. However, little is known about the cognitive capacity of the care team to deliver quality and timely care services, and the operational and patient factors that lead to cognitive overload, negatively impacting decision making [4]. Both the intense physical task load (e.g., frequent bedside visits, invasive interventions), and the increasing amount of patient data and information add cognitive burden to the care team. Patient data and information are critical as they serve as the input for clinical decision making; however, they could paradoxically hinder the team’s cognitive function [5, 6]. As the care team reaches the cognitive capacity limit, the quality and the timeliness of the clinical decisions might be impaired, which could result in diagnostic delays and errors [7–12]. The COVID-19 pandemic has further exacerbated the risk as many patients with complex conditions are admitted into ICUs, and hospitals have been struggling to expand ICU capacity to accommodate the pandemic demand surge [13, 14].

Systems engineers have developed frameworks, e.g., Failure Modes and Effects Analysis (FMEA) [15], and Systems Engineering Initiative for Patient Safety (SEIPS) [16], to analyze systems and system failures. FMEA is a risk assessment method for identifying and mitigating potential failures in systems. SEIPS is a framework for understanding the structures, processes, outcomes, and their relationships in healthcare systems. These frameworks facilitate the investigation of complex adaptive systems that consist of convoluted interactions among system components over time. For example, FMEA has been utilized to analyze systems across industries including healthcare [17, 18] and SEIPS has recently drawn much attention to investigating post-pandemic healthcare systems [19, 20]. Inspired by the SEIPS framework, our central hypothesis is that clinical decision making in ICUs by the multidisciplinary care team can be modeled as a distributed cognitive system [21].

Extant studies have attempted to examine ICU system factors and cognitive load in qualitative and quantitative ways. Several measurements of workload have been proposed, including the therapeutic intervention scoring system [22], the NASA Task Load Index (TLX) [23, 24], and the electronic order volume in electronic medical records (EMR) [25]. Factors such as the assessment of new patients [26–28] and patients’ severity of illness [29, 30], and their impact on cognitive burdens and workload have been investigated. These studies were based on a small sample of patients, which led to a low statistical power, and the time-dependency property of system factors was not considered. Overall, there is a lack of understanding regarding the factors associated with the cognitive overload.

The overarching goal of our research is to build a model to represent the distributed cognitive network behavior of an ICU team and to use it to explore the capacity limits of a team of clinicians under a variety of operational conditions. In so doing, we sought to highlight system vulnerabilities and failure points that might exist in ICUs. To achieve this, in this study, we developed a conceptual framework, and used data from the Mayo Clinic EMR [31] to further investigate the relationship between workload factors and the output of decision making described in the conceptual framework. The workload factors include patient census, rate of admissions, and key patient characteristics (unit census, rate of new admissions, patient severity of illness, number of ventilated patients, and mortality risk). For the output of the care team’s decision making, the number of medication orders was chosen based on the experience of a team of clinical experts.

Orders are a unit of productivity of the multidisciplinary care team and a surrogate for distributed cognitive function [32–35]. The generation of orders in high-acuity environments requires demanding cognitive processes, and the efforts are typically made within a limited time span by the whole team. Absorbing a large amount of patient data and information imposes cognitive burdens on the care team. Additionally, interruptions that frequently occur in ICU environments while generating orders impair working memory [36, 37]. Therefore, making large numbers of orders in a unit time implies a high cognitive load for the care team.

Among orders, medication orders are typically documented in the EMR and can be quantified with relatively good accuracy [38]. Compared to other types of orders (e.g., consultation, physical examination and procedures, labs, etc.), first, medication orders are relatively cognitively demanding tasks (beyond a simple button click); second, they exhibit a high generation frequency and a large volume (in contrast, procedures are ordered far less frequently) to ensure the power of statistical analysis. Lastly, we limited our focus on medication orders alone to minimize the confounding effect of different types of orders (e.g., differences in frequency, volume, and cognitive load by order types).

## Methods

### Setting and electronic environment

A retrospective observational study was conducted using EMR data collected from the medical ICU (MICU) at an academic medical center, Mayo Clinic in Rochester, MN, between January 2016 and April 2018. This study was approved as a minimal risk/exempt study by the Mayo Clinic institutional review board (IRB). The data-sharing agreement between the University of Florida and Mayo Clinic was also approved by the Mayo Clinic IRB.

The MICU under study is a 32-bed medical/respiratory intensive care unit which serves a wide variety of patient problems, including gastrointestinal bleeding and cardiovascular, metabolic, respiratory, renal, and multisystem failure [39]. We focused primarily on the MICU to avoid any confounding effects due to the differences in bed capacity, patient mix, and organizational factors like staffing models and workflow across units. During the study period, the MICU had an average daily admission rate of 9.0 (SD = 3.4) and a median midnight census of 18 (interquartile range (IQR) = 15–20) patients. Two ICU teams where each consists of a consultant who leads the team, an attending critical care specialist, supervising fellows, residents, and nurse practitioners provide care with two shifts. Their tasks include physical examination, chart review, plan of care discussion, invasive procedure, specialty consult, and patient/family communication. This staffing model was based on the institutional practice policy decision and was consistent throughout the course of the study period. The multidisciplinary ICU rounds typically occur every morning (8–10 am) with fellows, residents, and nurse practitioners, as well as nurses, respiratory therapists, a pharmacist, and an attending critical care specialist [39], and additionally multidisciplinary ICU rounds occur at 2 pm and 10 pm if needed.

### Participants

The participants were patients who had ever stayed in the MICU, including those transferred to and from the MICU, and those who stayed in the MICU exclusively, i.e., no transfer, during the study period. Based on this criterion, 6240 MICU admissions were included of which 4822 were unique patients.

### Data collection

EMR data were retrieved from the ICU datamart, which is a Microsoft SQL-based database that assembles a near-real-time copy of clinical and administrative data from the Mayo Clinic EMR [31]. Data were processed to remove any identifiable information. Specifically, to reserve the temporal relationship of data points, the actual dates were mapped to fake dates and the key was retained by the data custodian. The following three categories of data were primarily collected for this analysis: bed locations, patient outcomes, and medication orders. Bed locations consist of detailed bed sites during a patient’s hospitalization and their transfers between different units/floors. This information was utilized to capture the patient census, which is a time dependent variable. In addition to patients’ length of stay (LOS) and mortality outcome, we also extracted the features associated with the system workload, including invasive mechanical ventilation (IMV) usage, admission date and time, and daily Sequential Organ Failure Assessment (SOFA) scores [11, 12, 29, 30]. Medication orders were used as a surrogate marker of the output of the care team’s decision-making process. The order time and medication descriptions for each ICU stay were collected. We created a comprehensive sequential event (temporal) dataset, including the events of admission, discharge, transfer, and medication orders and their time stamps, for individual patients.

### Design framework

**Fig. 1:**
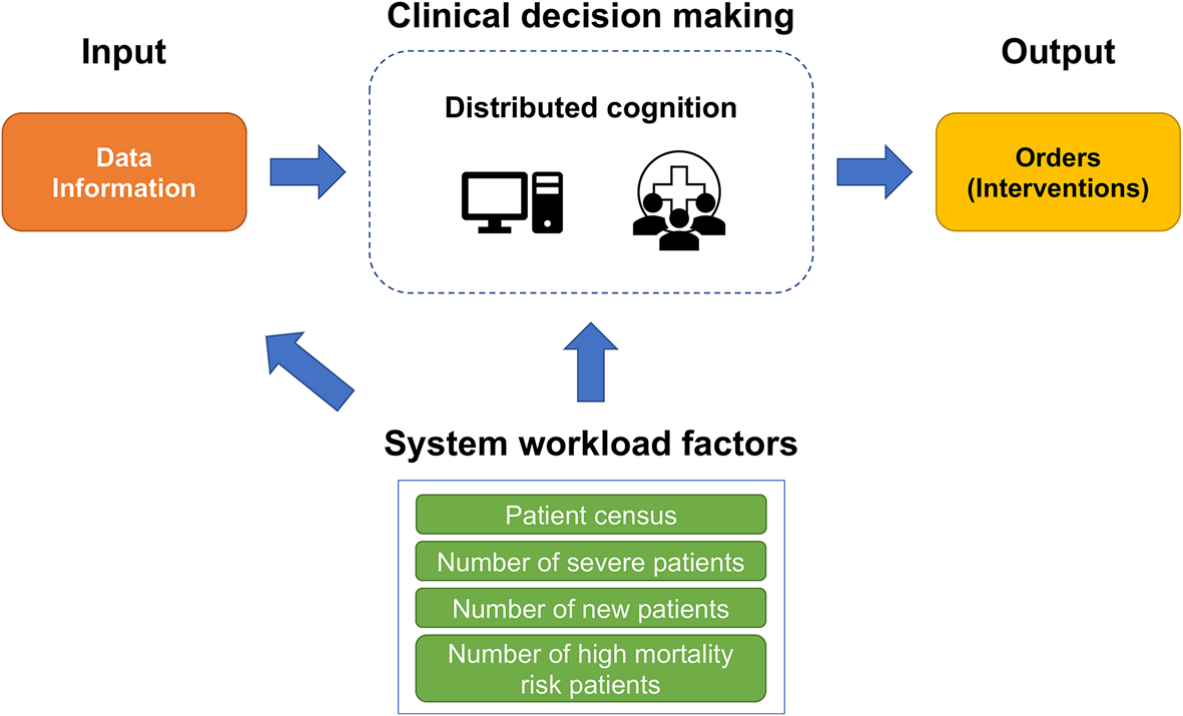
The conceptual framework of distributed cognition in ICUs. Information and data of patients are fed to the care team for clinical decision making. The distributed cognitive system includes both the care team and artifacts like technology. The output includes clinical interventions such as medication orders. The system workload factors (such as the number of patients and patient characteristics) affect the input (e.g., change in quantity) and subsequently affect the decision-making process (e.g., trigger cognitive overload)

The conceptual framework of a distributed cognitive system in ICUs is illustrated in Fig. 1. To prescribe medication orders, a multidisciplinary care team makes joint clinical decisions using data and information as the input. The patient data and information are obtained from bedside observation, EMR documentation, and communication among team members, as well as others. Since our focus was the relationship between system workload factors and the production of medication orders, we followed the SEIPS framework [16], a systems engineering approach to understanding the interactions between humans and the healthcare system. The following four factors were considered the system workload factors in this study: patient census and census of severely-ill patients, new patients, and high mortality risk patients. The patient census is a well-documented ICU capacity strain metric [8]. We further considered patients who had IMV during their stay as severe patients. The data constraints on us did not allow us to look at other markers of severity. However, the use of IMV is typically accurately documented in EMR and we also expect that the transitions in health states (which need IMV and other invasive interventions) are the major contributor to workload. Next, since the care team spends more time and efforts on newly admitted patients [27, 28], we marked patients within 3 h post-admission as “new patients.” Most of the care activities to admit and start therapeutic management of a patient require less than 3 h. After these activities are completed, patients are regarded as “regular patients.” Lastly, to measure the mortality risk of patients, we used daily SOFA scores, measured from 0 to 24 based on the degree of dysfunction of six organ systems [40]. The daily scores are calculated at admission and every 24 h by an automated SOFA scoring computer program [41]. For tractability, the mortality risk was treated as a static feature for patient classification purposes. We identified high mortality risk patients based on the patient’s daily SOFA score(s) with the following criteria: an initial SOFA score above 11, the highest daily SOFA score above 11, or an average of daily SOFA scores above 5, across their entire ICU stay. The cutoffs were chosen based on expert opinions and the literature [42]. Admittedly, many continuous and temporal data were reduced to categorical variables and some information might be neglected through this transition. However, this was necessary to enable a first-cut and tractable analysis at the system level. The robustness of these operations was examined (see the Sensitivity Analysis Section).

We aimed to test the hypothesis that, an ICU operational condition characterized by a high patient census, especially with the presence of large numbers of severe, new, and/or high mortality risk patients, will increase the risk of cognitive overload among the care team. This hypothesis is supported by evidence that decision-making worsens in strained ICUs [7–12]. Decisions like making prescriptions for severe, new, or high mortality risk patients can be more complicated and thus time and effort consuming. The increase in interruptions brought by the increase in patient census also disturbs decision making and imposes additional cognitive loads. Therefore, we examined a phenomenon that the number of medication orders per patient drops significantly when the patient census increases in the study setting. In other words, there exists a breakpoint where the number of medication orders starts to plateau and no longer increases at the same rate as the increase in patient census. This phenomenon implicitly suggests that the care team has reached their capacity cap.

### Statistical analysis

To test the hypothesis, univariate piecewise Poisson regression models were developed with hourly ICU patient censuses as the independent variable and the total number of medication orders per hour as the dependent variable. These models were chosen because the outcome is a discrete variable, i.e., a count. The coefficients obtained from each model measure the strength of association in different line segments. Then, these coefficients were compared using the generalized F-test. The null hypothesis was the coefficients of these line segments (representing the rate of medication orders generated per unit patient census) are identical. If the null hypothesis is rejected, we validate the cutoff as where the rate starts to change. Furthermore, we compared the number of medication orders per patient per hour before and after the identified cutoff using the two-sample t-test. Rare occasions (e.g., data of patient census below 10 and over 27; see Table S1 in Additional file 1) were excluded to gain test efficiency. We have conducted a sensitivity analysis to ensure that the removal of these points does not affect the main observations.

**Fig. 2:**
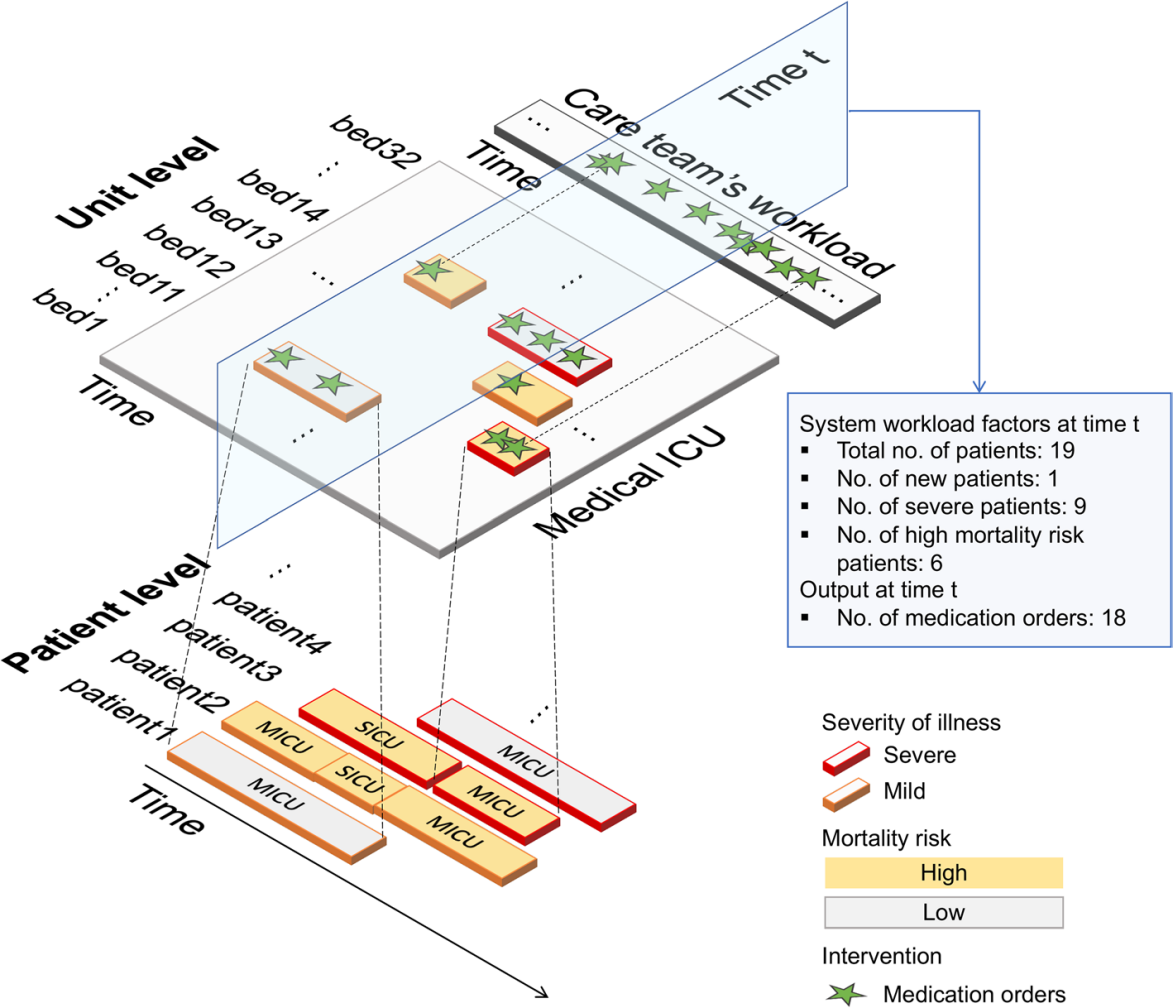
ICU system dynamics. The first layer describes individual patient stays in ICUs. ICU admission for each patient is represented by one or more box(es) depending on locations (bed sites) where they are taken care of. The width of the boxes reflects patients’ LOS and patients within the first 3 h post-admission are considered as new patients. Their severity of illness evaluated as non-severe (mild) or severe is differentiated by box border colors (orange and red, respectively). Also, filled colors indicate the mortality risk of patients: high (yellow) and low (gray). The second layer describes the dynamics of system workload factors and the output of medication orders in the MICU. Patients’ medication orders are marked as green stars. The system workload factors reflect the care team’s workload and cognitive load at the time point

To further characterize the patient census, at the time of interest, there could be a high (h) or low (l) presence of severe patients, new patients, and high mortality risk patients. Figure 2 exhibits the system-level dynamics that associate the system workload factors with medication orders. To simulate the COVID-19 pandemic surge, we modeled a scenario where the MICU has a high percentage of ventilated patients (i.e., a setting with more than 60% of the present patients having ever used IMV during their ICU stay). We compared the normal ICU setting with fewer IMV patients and the simulated pandemic ICU setting. To examine the effect of time, we categorized time into three windows, rounding (8:00 am to 9:59 am), daytime (10:00 am to 9:59 pm), and nighttime (10:00 pm to 7:59 am). Then, we developed multiple univariate piecewise Poisson regression models given each factor and followed the same test procedure.

## Results

Among 4822 patients, 869 patients had been admitted to the MICU multiple times, and a total of 6240 MICU hospitalizations was identified during this study period. We assumed that each hospitalization is independent because the patient characteristics in this study were time-varying and different, depending on their specific hospitalization. As described in Table 1, the median age was 64.9 (IQR = 51.8–76.7); male patients accounted for more than half of the population; the median LOS was 1.5 days (IQR = 0.9–2.7); 584 (9.4%) patients died during their stay; 1677 (26.9%) patients had used IMV during their stay and the median days on IMV were 1.1 (IQR = 0.4–3.0) among all patients.

**Table 1:** Demographics and hospitalization characteristics among 4822 unique MICU hospitalized patients and 6240 MICU hospitalizations during the study period

Demographics	Median (IQR) or n (%, N)
Age at admission	64.9 (51.8–76.7)
Male sex	2690 (55.8%, *N* = 4822)
Hospitalization characteristics	
LOS	1.5 (0.9–2.7)
Mortality	584 (9.4%, *N* = 6240)
IMV usage	1677 (26.9%, N = 6240)
Days on IMV	1.1 (0.4–3.0)
Initial SOFA scores	4 (2–7)
Highest SOFA scores	5 (3–7)
Average SOFA scores	3.7 (2–5.3)

All but sex are summarized based on hospitalizations because they are hospitalization-dependent

### System workload factors

The hourly patient census had a median of 18, with an IQR of 16–21. The average bed utilization was 56.3%, calculated by the median of daily patients over the bed capacity [43]. For severity of illness, the percentage of patients who had ever had IMV usage was measured on an hourly basis. The median was 45% (IQR = 36.8–54.2%). In terms of new admissions, the overall hourly admission rate is 0.37 (SD = 0.65), 0.42 (SD = 0.68) for rounding and daytime, and 0.31 (SD = 0.60) for nighttime, respectively. The census of new patients differed by the overall patient census. The censuses were higher, ranging from a total of 13.5–20 new patients per rounding and daytime, under a moderate bed occupancy (13–20 patients), and ranging from 7 to 11.5, under a high bed occupancy (21–26 patients). This is also true for the nighttime period. The censuses of new patients ranged from 11 to 16.3 per nighttime with a moderate bed occupancy (12–21 patients), and ranged from 5 to 9 with a high bed occupancy (22–27 patients), see Table S2 in Additional file 1.

For the mortality risk, the medians of the initial, highest, and the average daily SOFA scores were 5 (median, IQR = 3–8), 5 (median, IQR = 3–8), and 3.7 (median, IQR = 2–5.2), respectively. High mortality risk patients accounted for a median of 33% (IQR = 26.3–41.7%) for every hour.

### Medication orders

**Fig. 3:**
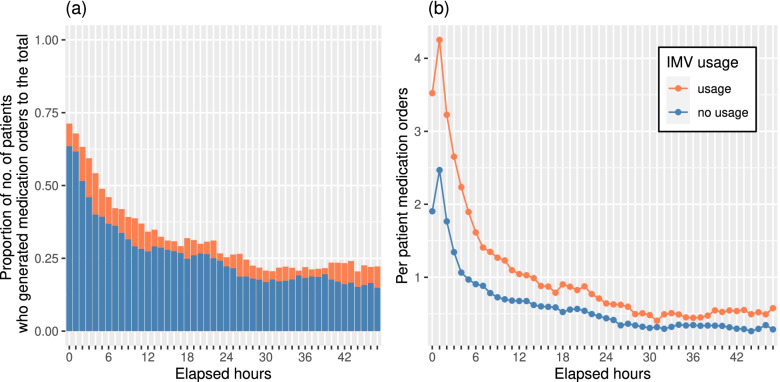
The number of medication orders generated during each hour of the first 48 h since ICU admission by IMV usage (orange for patients who had ever used IMV during their stay vs. blue for those who had not): **a** the percentage of patients who had generated medication orders, and **b** the hourly average of per patient medication orders for each elapsed hour

A total of 235,200 medication orders were recorded for these 4822 patients during their hospitalization. In the MICU, an average of 12.6 medication orders per hour was generated with a standard deviation (SD) of 9.6. The percentage of patients who had generated medication orders, and the per patient medication orders during each hour of the first 48 h are shown in Fig. 3(a) and (b), separated by IMV usage vs. no usage. Patients with IMV usage were more likely to generate medication orders compared to the contrast no IMV usage group, by 63% more on average. Compared to those with no IMV usage (hourly average = 0.7; SD = 1.8), the care team ordered more medications for patients with IMV usage (hourly average = 1.1; SD = 2.8) during the first 48 h of their admission (*p* < 0.001). For the comparison regarding new and regular patients, the per patient medication orders during the first 3 h were larger (hourly average = 2.1; SD = 3.5), as opposed to those of the following (hourly average = 0.7, SD = 1.6; *p* < 0.001). Those for the high and low mortality risk patients during the first 48 h were 1.1 (hourly average; SD = 2.5) and 0.8 (hourly average; SD = 1.8), *p* < 0.001. We only showed the statistics of the first 48 h, since the median patient LOS was 1.46 days (IQR = 0.84–2.72 d), i.e., the majority of patients were discharged after 48 h. The comparisons were summarized in Table 2. Overall, the number of medication orders per hour was dependent on IMV usage, new admission, and high mortality risk.

**Table 2:** Comparisons of the number of hourly medication orders by patient characteristics

Characteristics	Average (SD)	*P*
IMV usage		< 0.001
Usage	1.1 (2.8)	
Non-usage	0.7 (1.8)	
New patients		< 0.001
New	2.1 (3.5)	
Regular	0.7 (1.6)	
High mortality risk patients		< 0.001
High	1.1 (2.5)	
Low	0.8 (1.8)	

*P*-values were calculated using the two-sample t-test. If a *p*-value is less than 0.05, the two quantities are considered significantly different

### Association analysis

**Fig. 4:**
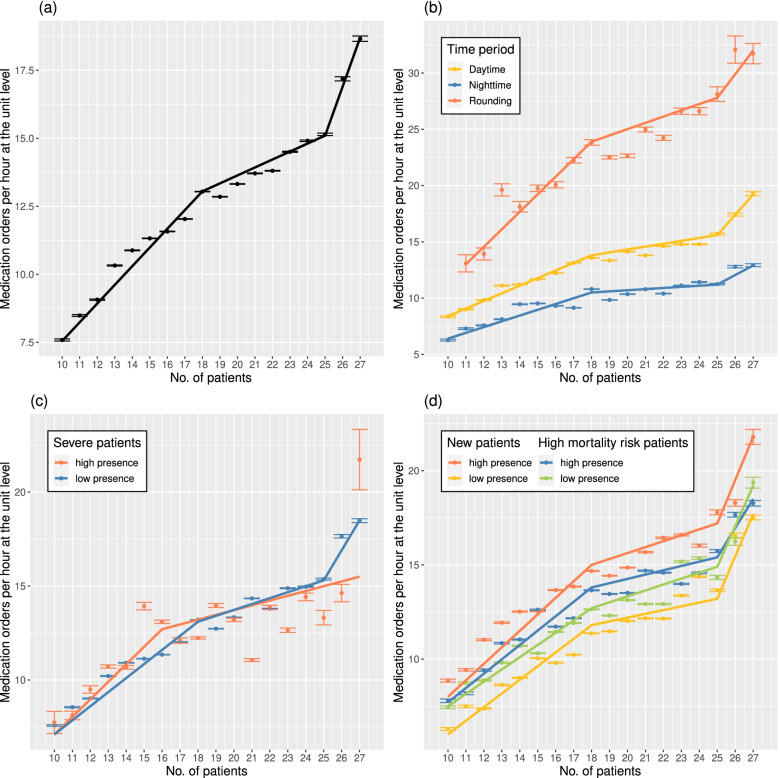
Medication orders per hour against patient census: **a** overall; **b** when controlling for the time periods (for the rounding period, samples with low and high census were excluded due to low sample size); **c** when controlling for the presence of severe patients (high vs. low); and **d** when controlling for the presence of new patients and high mortality risk patients, respectively. The high presence of severe patients was defined as an ICU operational condition with more than 60% of the present patients having ever used IMV at the moment. The high presence of new patients was defined as an ICU operational condition with more than one new patient at the moment. The high presence of high mortality risk patients was defined as an ICU operational condition with more than 33% of the present patients having higher SOFA scores than the chosen criteria at the moment. An error bar indicates a 95% confidence interval for the average of medication orders given a certain patient census

The statistical test results of the univariate piecewise Poisson regression were provided in Table S3 in Additional file 1. The relationship between patient census and the total number of medication orders (hourly) is illustrated in Fig. 4(a). Compared to a steep increase in orders when the census was below 18, the rate reduced between the range of 19–25, and then the rate was slightly recovered. The regression results validated that the two slopes before and after the breakpoint 18 were significantly different (*p* < 0.001). This suggests a reduction in medication orders per patient per hour when the patient census was above 18 patients, i.e., average = 0.74; SD = 0.56 vs. average = 0.65; SD = 0.48; *p* < 0.001, see Table 3. In short, the care team’s cognition function was impacted when the ICU capacity reached the 56% (18 out of 32) occupancy, and this phenomenon continued to around 80% (26 out of 32) of the ICU capacity. The curve rebounded when the census was high, possibly due to purposely restraining new admissions when the bed occupancy is high (see Table S1 in Additional file 1).

**Table 3:** Comparisons of medication orders per patient per hour before and after the cutoff

Characteristic		Sample size	Cutoff	No. of medication orders per patient per hour (average; SD)		*P*
				Patient census≤Cutoff	Patient census>Cutoff	
Overall		18,630	18	0.74(0.56)	0.65(0.48)	< 0.001
Time periods	Daytime	9294	18	0.79(0.53)	0.67(0.41)	< 0.001
	Nighttime	7784	18	0.61(0.47)	0.50(0.36)	< 0.001
	Rounding	1552	18	1.31(0.84)	1.15(0.71)	< 0.001
Severe patients	Low	16,252	18	0.74(0.56)	0.66(0.48)	< 0.001
	High	2378	16	0.81(0.59)	0.63(0.47)	< 0.001
New patients	Low	10,170	18	0.63(0.52)	0.59(0.47)	< 0.001
	High	8460	18	0.85(0.58)	0.74(0.48)	< 0.001
High mortality risk patients	Low	9623	18	0.72(0.56)	0.63(0.48)	< 0.001
	High	9007	18	0.77(0.56)	0.67(0.48)	< 0.001

The cutoff is the patient census where the rate of medication orders generated per patient started to change. The difference was tested by the two-sample t-test and the *p*-values are shown, for the overall (ungrouped) data, and subgroups, stratified by time and system workload factors, respectively. For workload factors, being “Low” indicates a low presence of the featured patients in the entire census. If a *p*-value is less than 0.05, the two quantities are considered significantly different

The same was true when controlling for time, as exhibited in Fig. 4(b). The statistical tests confirmed that the two pairs of slopes, measured with a census below 19 or above 18, were significantly different (*p* < 0.001 for the daytime and nighttime; and *p* = 0.032 for the rounding time as displayed in Table S3 in Additional file 1). Also, the number of medication orders per patient per hour significantly dropped (0.79 vs. 0.67, *p* < 0.001 for the daytime; 0.61 vs. 0.50, *p* < 0.001 for the nighttime; and 1.31 vs. 1.15, *p* < 0.001 for the rounding time, see Table 3). Thus, we concluded that time of the day affects the relationship between patient census and medication orders, but the same breakpoint exists regardless of time.

Next, the comparison between the normal ICU setting with fewer IMV patients, and the simulated pandemic ICU setting is exhibited in Fig. 4(c). It can be seen that the curve changes considerably when the ICU contained severely-ill, ventilated patients (indicated by IMV usage to match the feature of COVID patients). In the simulated ICU coping with patients with features resembling that of COVID patients, the rate of new medication orders fell significantly (*p* = 0.032, see Table S3 in Additional file 1), when the census went about 16 patients, and did not recover until the unit census stabilized above 26 patients. The number of medication orders per patient per hour was reduced from an average of 0.81 (SD = 0.59), to an average of 0.63 (SD = 0.47), *p* < 0.001, see Table 3. This is different from the regular scenario where the breakpoint was 18. In addition, a recovery was not witnessed, indicating that in circumstances where the presence of severe patients is high, the change occurred at a lower census and persisted.

An increased presence of high mortality risk patients (e.g., Do Not Resuscitate patients) alone is not likely a major cause of overloading the care team, as depicted in Fig. 4(d) The curve of the high presence setting, i.e., above median (33%) present patients were at high mortality risk, was close to that of a low presence setting, and their breakpoints were identical (*p* < 0.001 for the low census and *p* = 0.010 for the high census, see Table S3 in Additional file 1). Similarly, an increased presence of newly admitted patients was not identified as a significant risk factor for reduction in medication orders, i.e., affecting the location of the breakpoint, although it led to more medication orders as depicted in Fig. 4(d). To support this, we examined the location of the breakpoint regarding the number of new patients admitted hourly, considering more than one new admission (median) in an hour as a high number of new patients. The breakpoints were identical regardless of the census of new patients (*p* = 0.023 for the low census and *p* = 0.001 for the high census, see Table S3 in Additional file 1).

### Sensitivity analysis

We adjusted the definition of high mortality risk patients with different criteria to show the definition does not affect the results. For instance, we tested the model with patients having an initial SOFA score above 11 being defined as high mortality risk patients. Correspondingly, fewer patients in the ICU were considered as of high mortality risk. Using the median presence (8.3%; IQR = 4.5–14.3%) to dichotomize the ICU operational condition into a low or high presence of high mortality risk patients, we had the observation similar to the main analysis: the presence of high mortality risk patients still was not a remarkable factor of medication order reduction. The breakpoint was not changed when there was a high presence of high mortality patients, see Table S4 in Additional file 1. Another sensitivity analysis including rare patient census occasions (e.g., patient census ranging from 4 to 9 and 28–29, see Table S1 in Additional file 1) was performed and the same breakpoints were identified as summarized in Table S5 in Additional file 1.

## Discussion

### Methods

A comprehensive understanding of the system workload factors and their contribution to cognitive overload is essential to ensuring quality and timely critical care delivery, as well as improving patient outcomes. Our distributed cognition framework guided by systems approaches can help bridge this knowledge gap. Specifically, our proposed framework serves as a supplement to the existing human-subject research approaches (e.g., NASA-TLX [13, 14], Electroencephalography (EEG), Heart Rate Variability, Functional Near-Infrared Spectroscopy (fNIRS) [44]). There are several limitations to these approaches. For instance, for NASA-TLX, there may be biases from respondents [45]; EEG and fNIRS can accurately measure cognitive load, but usually in a laboratory environment. Although fiberless, wearable fNIRS has been developed to enable the experiment in the real world [46], it is unlikely to be testable in high-acuity ICU environments due to patient safety concerns. In contrast, our framework is entirely based on observational data collected from real-world ICUs, which makes a large-scale study feasible, less costly and time-consuming. In addition, our framework focuses on the distributed cognitive load, while the others are designed for measuring individual cognitive load and may require additional procedures to calculate the distributed cognitive load. Thus, although subject to several limitations to be elaborated in the Limitations section, this framework enables us to achieve an investigation of the distributed cognitive function using a large amount of real-world data. Following the framework, we identified surrogate markers in EMR with satisfactory data quality and quantity, and used them to identify important cutoffs to show when cognitive burden may potentially impact the team’s function.

### Results and system interventions

Our model suggests a relationship between operational conditions, patient factors, and team cognitive performance that warrants further study. The care team is likely to suffer a long-lasting cognitive overload with a high presence of severely-ill patients, represented by a significant reduction in their ability to make medication orders and no recovery of their cognitive capacity. According to the situational awareness theory [47, 48], the care team is less likely to retain high comprehension when suffering cognitive overload. Raising the awareness of the care team about the potential burnout and breakdown will enable them to perceive their situation and become more agile to reach the stage of projection, i.e., taking timely actions to avoid cognitive overload. The insights obtained from this study serve as a valuable guideline to mitigate the risk of cognitive overload.

One guideline is to strategically allocate additional clinical resources to mitigate the impact of workload on cognitive function. As the presence of large numbers of severe patients constrained the care team’s ability to provide clinical interventions, if such a work environment is long-lasting (e.g., the pandemic setting), the staffing model that works in the normal ICU environment might not be sufficient and a new staffing model (e.g., having an additional care team) might be required. In contrast, if the workload surge is temporary, for instance, a large number of patients were admitted or planned to be admitted during a six-hour shift, then, a teleICU consultant can be introduced as a backup consultant of the care team [49]. Consequently, the breakpoint might vanish or shift to a higher patient census. Overall, the appropriate multidisciplinary composition of teams needed in acute and strained surge environments should be carefully determined. For instance, there might be a difference between house staff and advanced practice provider staffing models (particularly in the setting of increasing protocolized care) [50, 51].

Another remedy is to streamline the admission process to avoid an increased number of new patients in particular time periods so the workload can be evenly distributed across the day. This can potentially be achieved by reengineering the discharge process so that a better patient room turn around can be achieved [52]. For instance, if the discharge order can be placed in the early morning, more patients can be admitted earlier in the day rather than being pushed to the late afternoon. Additionally, geographic cohorting of similar patients, particularly ICU patients, has been demonstrated to be associated with reduced interruptions and improved coordination of care [53].

Our study also promotes the potential benefit of healthcare information technology. A clinical decision support system (CDSS) can support quick decisions with less effort to achieve workload reduction [54]. We observed that a high mortality risk was not influential with respect to burdening the care team. It might be attributed to the wide adoption of the computerized physician order entry (CPOE) system, which provides an order set that covers a variety of common clinical tasks. The use of it effectively reduces cognitive workload and minimizes the time-consuming procedures (e.g., adjusting doses of long-standing medication orders) [5, 6]. Another example is discharge planning using data-driven risk prediction models. Artificial intelligence based CDSSs to predict a target event (e.g., readmission [55, 56], disease diagnosis [57–59]) have attracted growing attention. Customizable CDSSs to project the expected workload of the care team and allocate the resources properly to avoid potential burnout are urgently desired.

### Limitations

There are several limitations of this study. Although the average LOS of the study participants and the severity level were similar to the nationwide data [60], the patient cohort was mainly from ICUs in a single center (Mayo Clinic, Rochester MN). The findings might be affected by the organizational factors such as hospital- and unit-specific protocols (staffing model, seasonal variation), workflows (e.g., nighttime or weekend admission), and patient mix (characterized by demographic and socioeconomic features such as race, ethnicity, and insurance status). Therefore, a multi-center study with diverse populations is a useful and important future direction.

Using medication orders as a surrogate of productivity allowed us to develop the model because complete medication orders can be accessed from EMR and quantified with relatively good accuracy. We acknowledge that the number of medication orders does not necessarily equate with cognitive load, and future work is warranted to understand mechanistically if this reduction in medication orders is primarily due to cognitive overload or other reasons such as changes in prescribing efficiency. A future study that employs validated methods (e.g., collecting physiological signals) to measure the cognitive load needs to be conducted to rule out other possibilities. In addition, a drill down into the specific orders may be instructive. For instance, when a patient is put on a ventilator, the ventilator bundle order set can be used to deliver several orders with one click, and the effort involved in making orders varies based on the order type (e.g., a new order or a modification of an established order). However, this detailed information is currently not available to the research team, and as an exploratory effort, the use of quantities of medication orders has provided us new findings and motivated us to further explore this phenomenon. Potential considerations for future work include a full characterization of detailed medication order types, and the collective use of other interventions such as lab orders and procedures. Our study can benefit from this extension upon obtaining the relevant data.

To evaluate the severity of illness of patients and to simulate the pandemic operation conditions, we primarily considered the IMV usage. This was built on the fact that ventilated patients in general require a higher level of care by the care team. We did not assess additional organ support such as dialysis due to data limitation. Ideally, capturing the physiologic transition states of patients like from being unstable to stable (and back to unstable) will allow us to better capture the change in the team’s cognitive load and performance. An effort to highlight activities associated with a high cognitive load (e.g., intubation, resuscitation, additional organ support, etc.) should be pursued.

The regression models generally assumed that other factors between the independent and dependent variables had been sufficiently accounted for. However, this is typically difficult to be validated. For instance, the simulated COVID-19 operation condition is likely a best-case scenario, as we mainly considered the patient features like prolonged LOS and IMV usage. We did not factor other stressors a novel disease can place on the cognitive function of the team, such as unknown pathology and unfamiliar mental models of the disease, the training and learning needed for personal protection equipment use, the use of new drugs. The genuine team cognitive capacity is therefore likely to be much less than what we captured in the current model. In addition, the drop in medication orders could also be attributed to decision fatigue in addition to cognitive overload and warrants further investigation [61–64].

Another limitation of the study is the lack of data regarding the individual activities and tasks of multidisciplinary care team members. The impact of team composition on the cognitive function could not be analyzed but may also play an influential role. In the next steps, we seek for a better understanding of the circumstances in which team cognitive function begins to break down in the ICU.

Lastly, we would like to note that our main takeaway is the variations in medication order generation based on the system workload and no direct inferences can be made about the association of cognitive capacity with clinical outcomes such as mortality, ICU/hospital LOS, and discharge destination. There have been studies and evidence demonstrating an association between increased medication errors and increased clinician workload [7–12]. The increase in workload can potentially increase interruptions in tasks and result in failures to progress to the completion of the task and errors during the completion of the task [36, 37, 65]. Therefore, our future work aims to test the robustness of the relationship between orders and cognitive overload, the operational contributors to cognitive overload, and the significance of cognitive overload on important patient-centered outcomes such as LOS, discharge disposition, and the cost of care.

## Conclusions

In this study, we showed that the care team’s capacity to prescribe medication orders became constrained when the ICU was more than 50% occupied, and the level of impact was elevated when there was an increased presence of severe patients and/or newly admitted patients who demanded intense clinical interventions. The model implies that in a pandemic setting, the ICU care team is even vulnerable and at frequent risk for a cognitive breakdown due to both surging demands and the complex nature of COVID illness. This work also emphasizes the importance of increasing situational awareness of the ICU care team to detect and react when the cognitive function becomes vulnerable. System interventions (e.g., supplementary teleICU consultants, reengineered admission and discharge processes, and geographic cohorting) and CDSSs can potentially mitigate the risk of cognitive overload.

## Supplementary Information

**Additional file 1: **Includes frequency of patient censuses during the study period (**Table S1**); admission rates given patient census during rounding and daytime and during nighttime (**Table S2**); piecewise Poisson regression results (**Table S3**); sensitivity analysis with the different criterion of high mortality risk patients (**Table S4**); and sensitivity analysis including rare patient census occasions (**Table S5**).

## Data Availability

The datasets analyzed during the current study are not publicly available due to the protocol but are available from the corresponding author on reasonable request and with the permission of the permission of the Mayo Clinic IRB.

## Abbreviations

CDSS: clinical decision support system
CPOE: computerized physician order entry
EEG: electroencephalography
EMR: electronic medical records
FMEA: failure models and effects analysis
fNIRS: functional near-infrared spectroscopy
ICU: intensive care unit
IMV: invasive mechanical ventilation
IRB: institutional review board
IQR: interquartile range
LOS: length of stay
MICU: medical intensive care unit
SEIPS: systems engineering initiative for patient safety
SD: standard deviation
SOFA: sequential organ failure assessment
TLX: task load index
