# Supplementary material for: Investigating the cognitive capacity constraints of an ICU care team using a systems engineering approach
Source: BMC Anesthesiol. 2022 Jan 4;22:10. doi: 10.1186/s12871-021-01548-7 (PMC8724599; doi:10.1186/s12871-021-01548-7)
Supplement: Supplementary file 1 — Additional file 1: Includes frequency of patient censuses during the study period (Table S1); admission rates given patient census during rounding and daytime and during nighttime (Table S2); piecewise Poisson regression results (Table S3); sensitivity analysis with the different criterion of high mortality risk patients (Table S4); and sensitivity analysis including rare patient census occasions (Table S5). [file 12871_2021_1548_MOESM1_ESM.docx]

Table S1 Frequency of patient censuses during the study period.

| Patient census | Frequency | Patient census | Frequency |
| --- | --- | --- | --- |
| 4 | 1 (0.0%) | 17 | 1592 (8.6%) |
| 5 | 6 (0.0%) | 18 | 1765 (9.3%) |
| 6 | 6 (0.0%) | 19 | 1771 (9.3%) |
| 7 | 35 (0.2%) | 20 | 1688 (8.9%) |
| 8 | 73 (0.4%) | 21 | 1488 (7.8%) |
| 9 | 165 (0.9%) | 22 | 1346 (7.1%) |
| 10 | 291 (1.5%) | 23 | 1023 (5.4%) |
| 11 | 432 (2.3%) | 24 | 812 (4.3%) |
| 12 | 701 (3.7%) | 25 | 491 (2.6%) |
| 13 | 795 (4.2%) | 26 | 312 (1.6%) |
| 14 | 985 (5.2%) | 27 | 269 (1.4%) |
| 15 | 1304 (6.8%) | 28 | 113 (0.6%) |
| 16 | 1565 (8.2%) | 29 | 28 (0.1%) |

Table S2 Census of new patients given patient census for rounding and daytime and for nighttime.

| Patient census | Census of new patients | | | | | |
| --- | --- | --- | --- | --- | --- | --- |
|  | Rounding and daytime  (8:00 am to 9:59 pm) | | | Nighttime  (10:00 pm to 7:59 am) | | |
|  | Median | Q1 | Q3 | Median | Q1 | Q3 |
| 10 | 10.0 | 13.0 | 15.0 | 10.5 | 14.5 | 18.0 |
| 11 | 13.0 | 19.0 | 24.5 | 13.0 | 13.0 | 16.0 |
| 12 | 11.8 | 20.5 | 25.0 | 10.0 | 14.0 | 21.0 |
| 13 | 17.5 | 21.0 | 26.0 | 14.0 | 17.0 | 20.0 |
| 14 | 15.5 | 23.0 | 28.0 | 16.3 | 19.0 | 23.0 |
| 15 | 17.5 | 22.0 | 30.0 | 12.3 | 17.0 | 20.0 |
| 16 | 14.0 | 21.0 | 28.0 | 11.0 | 17.0 | 22.0 |
| 17 | 15.5 | 23.0 | 28.3 | 11.0 | 16.0 | 20.0 |
| 18 | 16.8 | 23.5 | 28.0 | 12.0 | 18.0 | 22.0 |
| 19 | 20.0 | 25.5 | 30.0 | 10.3 | 15.5 | 20.0 |
| 20 | 13.5 | 21.0 | 26.5 | 12.0 | 17.0 | 23.0 |
| 21 | 9.3 | 16.0 | 25.0 | 12.0 | 18.0 | 20.0 |
| 22 | 11.0 | 18.0 | 26.0 | 9.0 | 13.0 | 18.0 |
| 23 | 11.0 | 15.0 | 23.0 | 6.0 | 11.0 | 16.0 |
| 24 | 11.0 | 19.0 | 26.0 | 9.3 | 13.0 | 21.0 |
| 25 | 11.5 | 14.0 | 20.5 | 6.0 | 13.0 | 16.5 |
| 26 | 7.0 | 12.0 | 25.8 | 9.0 | 15.0 | 20.0 |
| 27 | 16.0 | 16.0 | 16.0 | 5.0 | 8.0 | 11.0 |

Patients within three hours post-admission were marked as new patients. The median of hourly patient censuses was obtained for each period and was rounded if it was not a natural number. For example, there are 20 patients between 8 am-12 pm, 16 patients between 12 pm-4 pm, and 19 patients between 4 pm-9:59 pm. Then, the rounded median of patient census is 18. In addition, new patients were counted for each period, and the quantities were aggregated by the patient censuses.

Table S3. Piecewise Poisson regression results.

| Characteristic | | Sample size | Cutoff | Intercept | Rate | | *P* |
| --- | --- | --- | --- | --- | --- | --- | --- |
|  |  |  |  |  | Patient census≤Cutoff | Patient census>Cutoff |  |
| Overall | | 18630 | 18 | 1.675 | 0.049 | 0.044 | <0.001 |
| Time periods | Daytime | 9294 | 18 | 1.767 | 0.046 | 0.042 | <0.001 |
|  | Nighttime | 7784 | 18 | 1.562 | 0.043 | 0.037 | <0.001 |
|  | Rounding | 1552 | 18 | 2.129 | 0.056 | 0.050 | 0.032 |
| Severe patients | Low | 16252 | 18 | 1.646 | 0.050 | 0.046 | <0.001 |
|  | High | 2378 | 16 | 1.868 | 0.041 | 0.033 | 0.032 |
| New patients | Low | 10170 | 18 | 1.420 | 0.055 | 0.052 | 0.023 |
|  | High | 8460 | 18 | 1.828 | 0.048 | 0.043 | 0.001 |
| High mortality risk patients | Low | 9623 | 18 | 1.584 | 0.053 | 0.047 | <0.001 |
|  | High | 9007 | 18 | 1.782 | 0.044 | 0.041 | 0.010 |

The rate represents the exponential growth of medication orders per census. The difference between the two rates before and after the cutoff was tested and the *p*-values are shown, for the overall (ungrouped) data and subgroups stratified by time and system workload factors, respectively. For workload factors, being “Low” indicates a low presence of the featured patients in the entire census. If a *p*-value is less than 0.05, the two rates are considered as significantly different.

Table S4. Sensitivity analyses using a different criterion of defining high mortality risk patients.

| High mortality risk patients | Sample size | Cutoff | Intercept | Rate | | *P* | No. of medication orders per patient per hour (average; SD) | | *P* |
| --- | --- | --- | --- | --- | --- | --- | --- | --- | --- |
|  |  |  |  | Patient census ≤Cutoff | Patient census >Cutoff |  | patient census ≤Cutoff | patient census >Cutoff |  |
| Low | 9472 | 18 | 1.710 | 0.045 | 0.042 | 0.045 | 0.730(0.558) | 0.639(0.473) | <0.001 |
| High | 9158 | 18 | 1.658 | 0.052 | 0.046 | <0.001 | 0.759(0.559) | 0.659(0.479) | <0.001 |

Table S5. Sensitivity analyses including rare patient census occasions.

| Characteristic | | Sample size | Cutoff | Intercept | Rate | | p |
| --- | --- | --- | --- | --- | --- | --- | --- |
|  |  |  |  |  | Patient census≤  Cutoff | Patient census>  Cutoff |  |
| Overall | | 19057 | 18 | 1.666 | 0.049 | 0.045 | <0.001 |
| Time periods | Daytime | 9528 | 18 | 1.747 | 0.048 | 0.042 | <0.001 |
|  | Nighttime | 7941 | 18 | 1.555 | 0.043 | 0.037 | <0.001 |
|  | Rounding | 1588 | 18 | 2.182 | 0.053 | 0.047 | 0.049 |
| Severe patients | Low | 16645 | 18 | 1.645 | 0.050 | 0.046 | <0.001 |
|  | High | 2412 | 16 | 1.822 | 0.044 | 0.035 | 0.015 |
| New patients | Low | 9876 | 18 | 1.387 | 0.057 | 0.053 | 0.006 |
|  | High | 9181 | 18 | 1.840 | 0.047 | 0.043 | <0.001 |
| High mortality risk patients | Low | 10398 | 18 | 1.601 | 0.052 | 0.047 | <0.001 |
|  | High | 8659 | 18 | 1.757 | 0.046 | 0.042 | 0.003 |
